# Tracking a recent horizontal transfer event: The *P*-element reaches Brazilian populations of *Drosophila simulans*

**DOI:** 10.1590/1678-4685-GMB-2019-0342

**Published:** 2020-05-18

**Authors:** Ana M.L. Nascimento, Bráulio S.M.L. Silva, Marta Svartman, Gustavo C.S. Kuhn

**Affiliations:** 1 Universidade Federal de Minas Gerais, Departamento de Genética, Ecologia e Evolução, Belo Horizonte, MG, Brazil

**Keywords:** Horizontal transfer, *P*-element, transposable elements, Drosophila simulans

## Abstract

The “cut-and-paste” *P*-element present in some Diptera illustrates two important transposable elements abilities: to move within genomes and to be transmitted between non-mating species, a phenomenon known as horizontal transposon transfer (HTT). Recent studies reported a HTT of the *P*-element from *Drosophila melanogaster* to *D. simulans. P*-elements first appeared in *D. simulans* European samples collected in 2006 and spread across several populations from Europe, Africa, North America and Japan within seven years. Nevertheless, no *P*-element was found in South American populations of *D. simulans* collected between 2002 and 2009. We investigated the presence of the *P*-element in *D. simulans* collected in five Brazilian localities between 2018 and 2019, using a combination of methodologies such as PCR, DNA sequencing and FISH on chromosomes. Our experiments revealed the presence of the *P*-element in all sampled individuals from the five localities. The number of *P*-elements per individual varied from 11 to 20 copies and truncated copies were also observed. Altogether, our results showed that *P*-element invasion in *D. simulans* is at an advanced stage in Brazil and, together with other recent studies, confirms the remarkable rapid invasion of *P*-elements across worldwide *D. simulans* populations.

Transposable elements (TEs) are DNA sequences usually less than 15 kb long that possess an intrinsic ability to mobilize and change their genomic location, with new copies generated during the process. They are abundant components of eukaryote genomes and play an important role in generating genetic variation that impacts the evolution of the species in which they transit (Kidwell and Lisch, 2001; Schaack *et al.*, 2010; Bourque *et al.*, 2018).

The *P*-element is a well-studied eukaryotic transposable element in Diptera (reviewed by Rio, 1991; Clark *et al.*, 1994; Castro and Carareto, 2004). It is a “cut-and-paste” DNA transposon that excises as double strand DNA and reinserts elsewhere in the genome. Full autonomous elements are approximately 2,900 bp long but several copies may contain deletions and therefore be either non-autonomous or completely inactive. The *P*-element illustrates two important features of transposable elements: their ability to jump within genomes and their capacity of spreading between non-mating species, a phenomenon known as horizontal transposon transfer (HTT) (Silva and Kidwell, 2000).

When TEs invade a new species, they are rapidly amplified and cause a broad spectrum of effects (reviewed by Schaack *et al.*, 2010). At a later stage, the transposition activity is regulated or suppressed by several mechanisms that lead to a decrease in transposition rate, accumulation of mutations, excision and eventually extinction, unless a HTT event introduces the TE in a new species, leading to its persistence over time (Loreto *et al.*, 2008; Schaack *et al.*, 2010). Therefore, HTT is considered an important event within the life cycle of TEs and consequently for the evolution of genomes and species.

The first reported case of HTT in *Drosophila* involved the transfer of the *P*-element from *Drosophila willistoni* to *D. melanogaster*, an event that probably took place in the American continent around the 1950s (Anxolabéhère *et al.*, 1988). After this invasion, *P*-elements rapidly amplified intra-genomically and spread through *D. melanogaster* worldwide natural populations within five decades (Anxolabéhère *et al.*, 1988). As a further evidence of a recent HTT, the *P*-element from *D. willistoni* is nearly identical (only a single nucleotide difference) to that of *D. melanogaster*.

Remarkably, a second invasion has been recently reported, this time involving a *D. melanogaster P*-element variant into *Drosophila simulans*, in which no *P*-elements had previously been detected (Kofler *et al.*, 2015). *D. melanogaster* and *D. simulans* are closely related species from the *melanogaster* subgroup and are separated by approximately ∼5.4 Mya from their common ancestor (Tamura *et al.*, 2004). Both species share common ecological habits, similar geographic distribution and insertions from different TE families (Sánchez-Gracia *et al.*, 2005; Bartolomé *et al.*, 2009). The *P*-element that invaded *D. simulans* differs from the most common *D. melanogaster* variant by a nucleotide substitution at position 2040 (G → A) in intron 3. This substitution is also found in *D. melanogaster*, but at very low frequencies (0.16–2%) (Kofler *et al.*, 2015; Yoshitake *et al.*, 2018).

This second *P*-element invasion is supposed to be recent, since it was first detected in *D. simulans* collected in Europe only from 2006 on. In the following seven years, it rapidly spread across several *D. simulans* populations from Europe, Africa, North America and Japan (Hill *et al.*, 2016; Yoshitake *et al.*, 2018). The exact mechanism responsible for this HTT has not been discovered yet. In South America, the presence of *P*-elements has been previously investigated in *D. simulans* from a small sample of flies collected between 2002 and 2009 from one locality each in Brazil (Ubatuba = 9 flies), Peru (Cusco = 1 fly) and Colombia (Guaymaral = 2 flies) (Hill *et al.*, 2016). Although none of these localities showed flies with *P*-elements, it cannot be ruled out the possibility that *P*-elements were not detected due to the limited sampling.

In order to investigate whether the *P*-element reached South American *D. simulans* populations, we looked for its presence in flies recently collected in Brazil. Collection trips using conventional banana bait traps were made between June 2018 and January 2019 in five Brazilian localities: Ubatuba (SP01), São Carlos (SP02), Sorocaba (SP03), Serra do Cipó (MG01) and Caldas Novas (GO01) (Figure 1 and Table 1). *D. simulans* identification was based on morphological characters and confirmed by PCR using the primers (cidsimF 5′ GGTATTATTTGCTTTGCTTG 3′ and cidsimR 5′ CTGAGCTACCATTCGTTGG 3′) that we designed to specifically amplify a fragment from the *Cid* gene (*Centromere Identifier*) of *D. simulans* (Figure S1).

**Figure 1:**
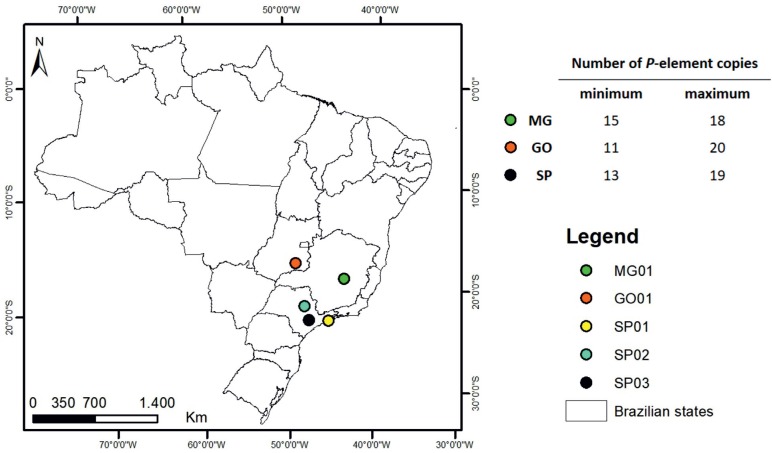
Approximate geographic locations of the Brazilian *D. simulans* populations studied. The table on the right shows the number of *P*-element copies estimated for three locations studied.

**Table 1:** Brazilian *Drosophila simulans* populations analysed for *P*-element presence.

Number of analysed specimens	Location and code	Coordinates	Date
6	Serra do Cipó, MG (MG01)	19°17′29.9″S 43°33′34.9″W	06/18/2018
6	Caldas Novas, GO (GO01)	17°39′40.6″S 48°44′30.6″W	07/25/2018
3	Ubatuba, SP (SP01)	23°32′35.9″S 45°13′56.1″W	01/28/2019
6	São Carlos, SP (SP02)	22°00′53.9″S 47°54′03.8″W	07/26/2018
4	Sorocaba, SP (SP03)	23°29′26.3″S 47°25′24.1″W	01/28/2019

We tested the presence of the *P*-element in three to six individuals from each locality. PCR was performed with oligos that amplify *P*-element exons 0, 1, 2, and 3, as described by Hill *et al.* (2016). Amplicons corresponding to all four *P*-element exons were visualized in all sampled individuals (Figure S2). In order to further confirm the G → A substitution present in *D. simulans P*-element copies, we PCR-amplified and sequenced a pool of non-cloned intron 3 sequences from individuals representing all five localities (see Internet Resources Section for chromatogram files). A sequence alignment containing the intron 3 sequences from our *D. simulans* samples together with a reference P-element from *D. melanogaster* (Flybase ID: FBte0000037) showed that all *D. simulans P*-elements share the nucleotide A at position 2040 (Figure 2), which is considered as a marker of this *P*-element invasion in *D. simulans* (Kofler *et al.*, 2015; Yoshitake *et al.*, 2018). In summary, the PCR and sequencing experiments showed that the *P*-element is present in all *D. simulans* sampled individuals from the five studied Brazilian localities.

**Figure 2:**
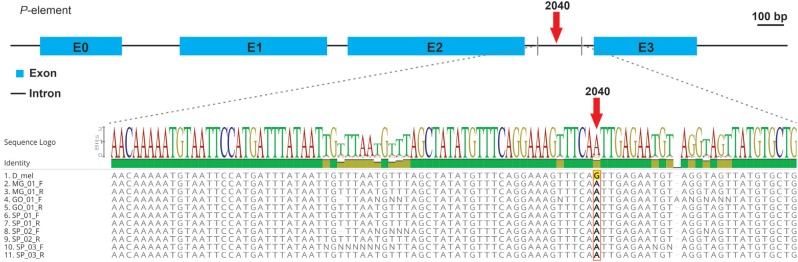
Schematic representation of a full *P*-element sequence (above). Nucleotide alignment containing partial intron 3 sequences from the five *D. simulans* sampled populations together with the reference sequence (Flybase ID: FBte0000037) from *D. melanogaster* (D_mel) (below). Position 2040 (highlighted) has the nucleotide A in all *D. simulans* sequences, which is a marker of this species' *P*-element. DNA sequencing was performed with Forward (F) and Reverse (R) primers directly from non-cloned PCR products. ‘N’ denotes nucleotide positions with low sequencing quality in the chromatograms.

In order to access the abundance of *P*-elements in *D. simulans* from Brazil, we performed double-FISH on polytene chromosomes, using as probes a 551 bp segment of exon 1 and a 662 bp fragment from exon 2. FISH experiments were conducted as described in Dias *et al.* (2014). A total of 15 larvae from three isofemale lines (DS-MG01, DS-GO01, DS-SP03) representing three localities (Serra do Cipó, Caldas Novas and Sorocaba) were analysed. The FISH experiments revealed *P*-element copies distributed along several euchromatic loci (see Figure 3). Despite the fact that previous studies suggested a preferential insertion of *P*-elements at subtelomeric regions (Karpen and Spradling, 1992; Kofler *et al.*, 2018), we detected only two signals at this location (Figure 3). The two probes co-hybridized except in a few cases in which only one probe hybridized indicating the existence of divergent or truncated *P*-element copies (Figure 3). Because the *P*-element invaded *D. simulans* very recently and in a single horizontal transfer event, all copies are very homogeneous (Kofler *et al.*, 2015). On the other hand, *P*-elements with internal deletions were reported in *D. simulans* experimental populations after 20 generations following their genomic spread from a single copy (Kofler *et al.*, 2018), while in natural populations *P*-elements with at least one missing exon were reported (Hill *et al.*, 2016). The presence of truncated copies is not uncommon, but their detection in our *D. simulans* samples is important because they can repress the mobilization of *P*-elements through the expression of nonfunctional transposases that compete with functional ones for binding sites (Lee *et al.*, 1998; Kofler *et al.*, 2018).

**Figure 3:**
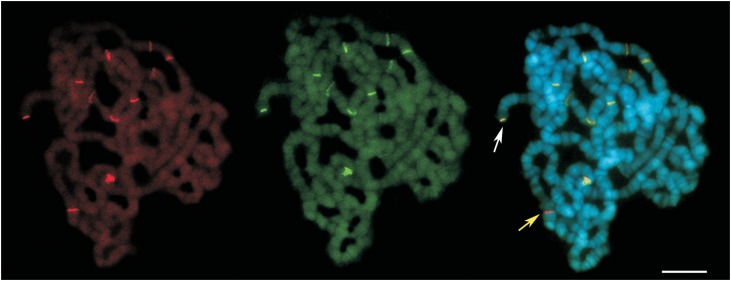
FISH on polytene chromosome of *D. simulans* (locality GO01) using two *P*-element probes: exon 1-red (left panel), exon 2-green (middle panel), and exon 1 + 2 (right panel). The chromosome was counterstained with DAPI-blue. Both arrows point to terminal regions insertions. The white arrow indicates co-hybridization of exon 1 and exon 2 probes. The yellow arrow shows a single probe hybridization (exon 1). Scale bar represents 10 μM.

Based on the signals revealed by our FISH experiments, the number of *P*-element copies in three Brazilian sampled populations varied from 11 to 20 copies per individual. However, these numbers should be taken as underestimates, because the same hybridization signal may contain more than one copy or some copies may lack the two exons segments used as probes.

We also investigated the *P*-element presence in additional species captured with *D. simulans* at the same bait, but without previous record of *P*-elements in their genomes. The PCR using oligos for *P*-element exons 0, 1, 2, and 3 produced no amplicons in all tested species: *Zaprionus indianus, D. nasuta, D. malerkotliana, D. mediopicta, D. ananassae, D. mirim* and one species from the *Cardini* group, all collected in 2019 (Figure S3). Whereas this result is insufficient to draw general conclusions, it may be useful as a reference for future studies tracking *P*-element invasions in other *Drosophila* species. In fact, Serrato-Capuchina *et al.* (2018) reported that *P*-elements also recently invaded *D. yakuba*, another species from the *melanogaster* group.

In conclusion, we provide the first report on the presence of the *P*-element in South American populations of *D. simulans*. This element has not been previously detected in samples collected between 2002 and 2009, but was found in specimens from five localities collected between 2018 and 2019. It is worth mentioning that *P*-elements were not detected in flies collected in Ubatuba in 2004 (Hill *et al.*, 2016), but in our study all sampled individuals from this locality presented the *P*-element, just 14 years later. Altogether, our results showed that this invasion is at an advanced stage and, together with other recent studies, confirms the trend of a remarkable rapid spread of *P*-elements across worldwide populations of *D. simulans*.

## Supplementary material

The following online material is available for this article:

Figure S1 - Agarose gel (1.5%) showing the PCR products with oligos (in red) designed to amplify a region of the *Cid* gene from *D. simulans* but not from *D. melamogaster*, as shown.

Figure S2 - Agarose gel (1.5%) showing the PCR products from *D. simulans* using oligos designed to amplify P-element exons 0, 1, 2 and 3, as described by Hill *et al.* (2016).

Figure S3 - Agarose gel (1.5%) showing the PCR products from several species captured with *D. simulans* at the bait, using oligos designed to amplify P-element exons, 1, 2 and 3, as described by Hill *et al.* (2016).
