# Supplementary material for: Tracking a recent horizontal transfer event: The P-element reaches Brazilian populations of Drosophila simulans
Source: Genet Mol Biol. 2020 May 18;43(2):e20190342. doi: 10.1590/1678-4685-GMB-2019-0342 (PMC7236489; doi:10.1590/1678-4685-GMB-2019-0342)
Supplement: Supplementary file 1 [file 1415-4757-GMB-43-2-e20190342-s1.pdf]

# Supplementary material to Tracking a recent horizontal transfer event: The *P*-element reaches Brazilian populations of *Drosophila simulans*

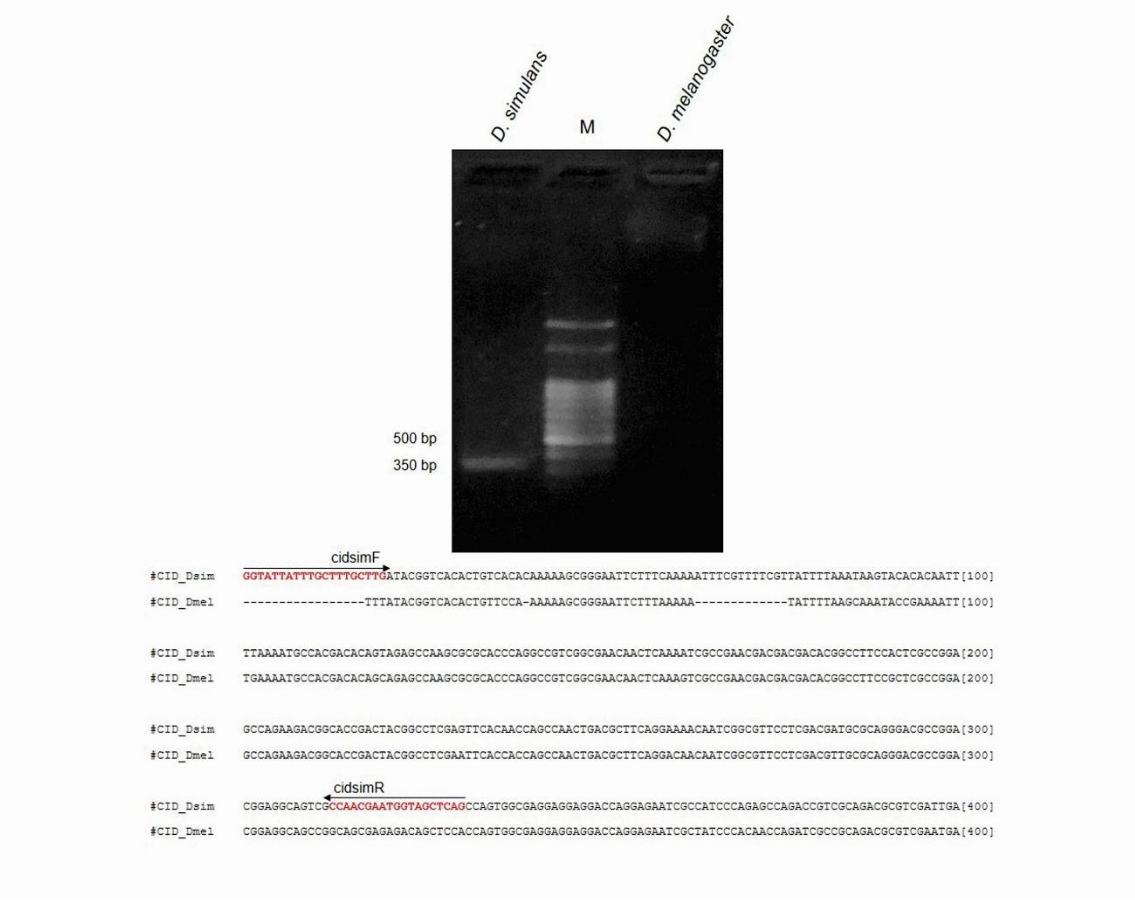


**Figure S1** - Agarose gel (1.5%) showing the PCR products with oligos (in red) designed to amplify a region of the *Cid* gene from *D. simulans* but not from *D. melamogaster*, as shown. M=1Kb plus DNA Ladder.
