# Supplementary material for: Tracking a recent horizontal transfer event: The P-element reaches Brazilian populations of Drosophila simulans
Source: Genet Mol Biol. 2020 May 18;43(2):e20190342. doi: 10.1590/1678-4685-GMB-2019-0342 (PMC7236489; doi:10.1590/1678-4685-GMB-2019-0342)
Supplement: Supplementary file 2 [file 1415-4757-GMB-43-2-e20190342-s2.pdf]

Supplementary material to Tracking a recent horizontal transfer event: The *P*-element reaches Brazilian populations of *Drosophila simulans*

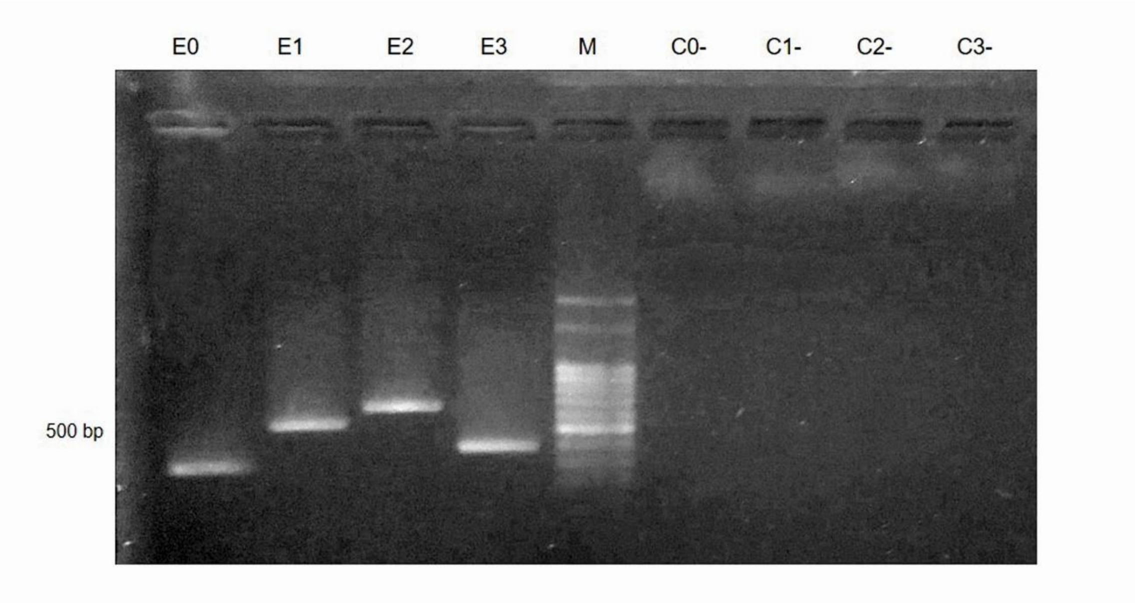

Figure S2 - Agarose gel (1.5%) showing the PCR products from *D. simulans* using oligos designed to amplify P-element exons 0, 1, 2 and 3, as described by Hill *et al* (2016). Negative controls (no DNA) are also showed. The pattern shown was observed for all *D. simulans* individuals sampled in our study. M=1Kb plus DNA Ladder.
