# Supplementary material for: Tracking a recent horizontal transfer event: The P-element reaches Brazilian populations of Drosophila simulans
Source: Genet Mol Biol. 2020 May 18;43(2):e20190342. doi: 10.1590/1678-4685-GMB-2019-0342 (PMC7236489; doi:10.1590/1678-4685-GMB-2019-0342)
Supplement: Supplementary file 3 [file 1415-4757-GMB-43-2-e20190342-s3.pdf]

Supplementary material to Tracking a recent horizontal transfer event: The *P*-element reaches Brazilian populations of *Drosophila simulans*

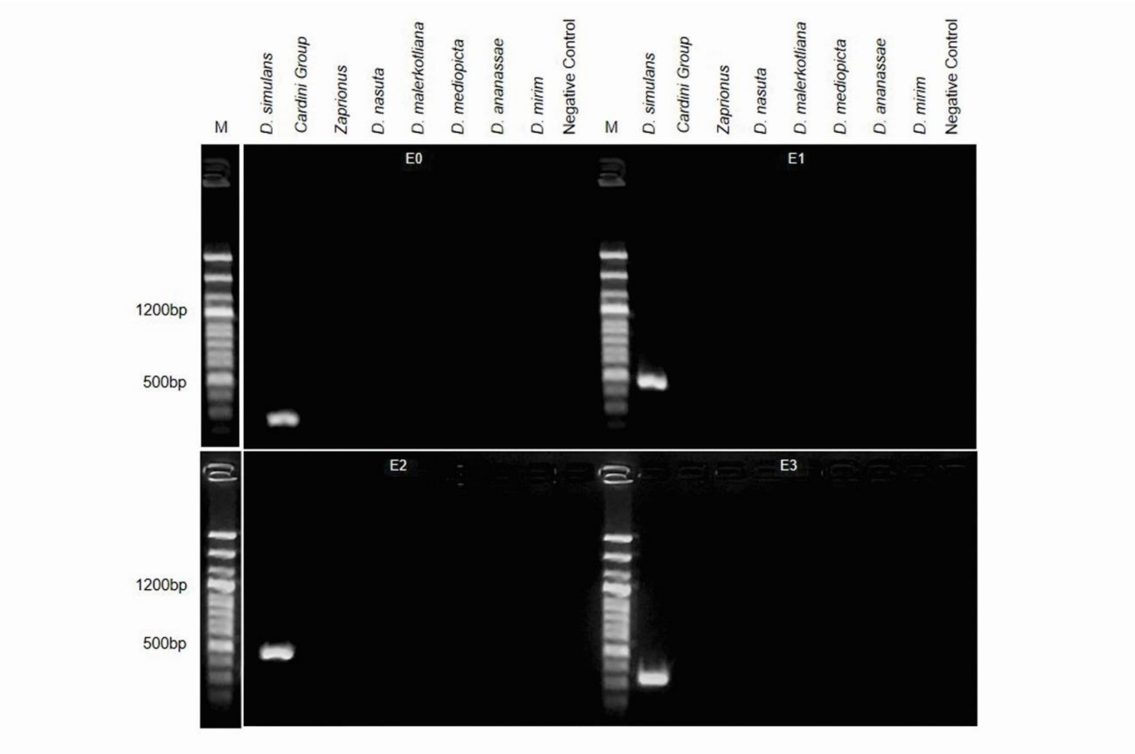

Figure S3 - Agarose gel (1.5%) showing the PCR products from several species captured with *D. simulans* at the bait, using oligos designed to amplify P-element exons, 1, 2 and 3, as described by Hill *et al.* (2016). Amplification was observed only for *D. simulans*. M = 1Kb plus DNA Ladder.
